# Inositol supplementation and body mass index: A systematic review and meta‐analysis of randomized clinical trials

**DOI:** 10.1002/osp4.569

**Published:** 2021-10-22

**Authors:** Meysam Zarezadeh, Azadeh Dehghani, Amir Hossein Faghfouri, Nima Radkhah, Mohammad Naemi Kermanshahi, Fatemeh Hamedi Kalajahi, Niyaz Mohammadzadeh Honarvar, Zohreh Ghoreishi, Alireza Ostadrahimi, Mehrangiz Ebrahimi Mamaghani

**Affiliations:** ^1^ Student Research Committee Tabriz University of Medical Sciences Tabriz Iran; ^2^ Department of Clinical Nutrition Faculty of Nutrition and Food Science Nutrition Research Center Tabriz University of Medical Sciences Tabriz Iran; ^3^ Department of Community Nutrition Faculty of Nutrition and Food Science Nutrition Research Center Tabriz University of Medical Sciences Tabriz Iran; ^4^ Department of Cellular and Molecular Nutrition School of Nutritional Sciences and Dietetics Tehran University of Medical Sciences Tehran Iran; ^5^ Department of Biochemistry and Diet Therapy School of Nutrition and Food Science Tabriz University of Medical Sciences Tabriz Iran

**Keywords:** body mass index, inositol, meta‐analysis, obesity, systematic review

## Abstract

**Background:**

Inositol is a sugar‐alcohol and recognized as a key component of cell membrane phospholipids. It has crucial role in the cell signaling pathways and contribute to improving glycemic responses. Although some earlier studies have revealed the effect of inositol mediating glucose uptake by improving insulin sensitivity, the benefit of inositol supplementation in patients with overweight and obesity is not completely understood. This study aimed to assess the impact of inositol supplementation on body mass index (BMI) through a systematic review and meta‐analysis of controlled clinical trials.

**Methods:**

A systematic search was performed to August 2021 in the following databases: PubMed‐Medline, Embase, Web of Science and Scopus. Fifteen controlled clinical trials investigating the effect of inositol on adult's BMI were finally included in the study. A random‐effects model was employed to estimate the effect size. Subgroup analysis was performed by dose, duration, age, type of inositol. Meta‐regression was used to investigate presence of any linear relationship. Begg's and Egger's tests were carried out to detect small study effect.

**Results:**

The results of pooled analysis showed that inositol supplementation significantly decreased BMI scores (WMD = −0.41 kg/m^2^; 95% CI: −0.78, −0.04; *p* = 0.028). Subgroup analysis was performed to identify the source of heterogeneity among studies (*I* ^2^ = 73.9%, *p* < 0.001), demonstrating supplementation duration, baseline BMI, mean age of participants, type of inositol and dosage were potential sources of heterogeneity. The effect of intervention was more clinically significant in participants with polycystic ovary syndrome (PCOS) and overweight/obesity. Inositol in the form of myo‐inositol (MI) had stronger effect on BMI reduction.

**Conclusion:**

The meta‐analysis suggests that oral inositol supplementation has positive effect on BMI reduction. Inositol supplementation could be considered as an adjunct treatment to improve body mass index.

## 1 INTRODUCTION

Inositol (1,2,3,4,5,6‐cyclohexanehexol) is a sugar‐alcohol with six‐carbon ring structure and has nine stereoisomers in nature namely myo‐inositol (MI) and Di‐chiro‐inositol (DCI). In addition, there is a 3‐O‐methyl form of DCI (D‐Pinitol) which is a natural derivate of inositol.^1^ While there is an endogenous production from glucose‐6‐phosphate, the major part of inositol comes from the exogenous sources like citrus fruits, beans, artichokes and most of other plants.^2^ Inositol is mostly represented in the free form or phosphatidylinositol (PI) form from animal sources and in the InsP6 form from the plant sources.^3^ Two reactions are needed to produce MI from glucose‐6‐phosphate. First, isomerization of glucose‐6‐phosphate to inositol‐3‐phosphate by the NADH‐dependent, cytosolic inositol 3‐phosphate synthase; and second, dephosphorylation of inositol 3‐phosphate to MI by inositol‐monophosphatase (IMPase) for further use.^3^ It has been revealed that, dietary MI supplementation in vivo is beneficially associated with lipid metabolism, bone formation, skeletal muscle metabolism, reproduction, nerve function, and brain actions.^4^ Finally, MI is degraded in the kidney in mammalians.^5^ Inositol deficiency occurs when food‐dependent intake, de novo synthesis, and intestinal and cellular uptake are reduced and catabolism and excretion are increased.^6^

Despite wide clinical usages of MI, there is still scarce information on the MI safety and side effects. A review study that assessed the safety of MI supplementation in clinical and non‐clinical studies reported that MI caused mild gastrointestinal side effects in highest dose of MI (12 g/day).^7^ A review study concluded that 2 g/day MI twice a day is the best MI regimen to improve metabolic pathways.^8^ Italian Diabetes National Societies (SID) considered 4 g/day of MI as a therapeutic agent in the treatment of gestational diabetes mellitus.^8^ It must be noticed that inositol is categorized as generally recognized as safe (GRAS) supplement by the FDA.^9^

Inositol also is a key component of cell membrane phospholipids in the form of inositol triphosphate (IP3) playing a pivotal role in the G‐protein‐dependent pathways.^10^ The inositol‐dependent pathways transduce the signal of some hormones including vasopressin, thyrotropin‐releasing hormone (TRH), thyroid‐stimulating hormone (TSH), angiotensin II, and GnRH.^11^ Also, inositol acted as an osmolyte in some tissues, such as kidney medulla and brain, where osmolarity has a pivotal biological meaning.^12^ In addition, inositol (either in the DCI or MI isoform) has been reported to mediate glucose uptake by improving insulin sensitivity. Insulin‐mimetic effects of MI or its isomers seem to be derived from inositol phosphoglycan (IPG) which contains MI or DCI.^13^ IPG acts as a second messenger in insulin signaling pathways and plays a vital role in the activation of enzymes involved in glucose uptake and utilization.^14^ Different rates of IPG imbalance have been observed in patients with diabetes and obesity.^15^

Pinitol is recognized to be a mediator of the insulin signaling route and to be contained in glycosyl phosphatidil‐inositol protein anchors.^16^ Moreover, studies have reported an increase in the number and quality of oocytes (immature eggs in the ovary) following the use of MI supplementation in women who underwent in vitro fertilization.^17^

There are some nutritional supplements that are advantageous in weight loss. A relatively new supplement which has attracted a lot of attention MI and DCI.^18^, ^19^ Recently, several studies have reported the effectiveness of DCI and MI in improving the complications associated with obesity in patients with polycystic ovary syndrome (PCOS) as well as metabolic syndrome and those who are suffering from diabetes.^19^, ^20^, ^21^ It seems that inositol beneficial effect on obesity and the associated disorders may be related to its involvement in insulin signaling and improving insulin sensitivity.

Overall, the beneficial effects of inositol supplementation on BMI has not been completely understood and therefore, the purpose of this systematic review and meta‐analysis is to address this issue, discuss the possible mechanisms and open a new window for further studies.

## 2 METHODS

### 2.1 Search strategy

The current study was performed according to the guiding principle of PRISMA (Preferred Reporting Items for Systematic Reviews and Meta‐Analyses).^22^ The study was registered in the PROSPERO (Ref. code: CRD42021225794).

A systematic literature search in electronic databases was conducted on PubMed‐Medline, Embase, Web of Science, and Scopus databases by one author (M. Z.) from inception up to August 2021. The language was limited to English articles only. The following key search terms were used to search publications by medical subject headings (MeSH) and titles or abstracts: (((((((((((“randomized clinical trial”[Publication Type]) OR “random*”[Title/Abstract]) OR “placebo”[Title/Abstract]) OR “groups”[Title/Abstract]) OR “trial”[Title/Abstract]) OR “clinical trial”[Title/Abstract])) OR “supplementation” [Title/Abstract]) OR “controlled” [Title/Abstract])) AND (((((((“Body Mass Index”[Mesh]) OR “Body Weight”[Mesh]) OR “Waist Circumference”[Mesh])) OR (((((((((((((((((“body mass index”[Title/Abstract]) OR “BMI”[Title/Abstract]) OR “body mass”[Title/Abstract]) OR “fat mass”[Title/Abstract]) OR “body weight”[Title/Abstract]) OR “weight”[Title/Abstract]) OR “obesity”[Title/Abstract]) OR “weight loss”[Title/Abstract]) OR “weight reduction”[Title/Abstract]) OR “Obesity”[MeSH Terms]) OR “obesity, abdominal”[MeSH Terms]) OR “weight change”[Title/Abstract]) OR “waist circumference”[Title/Abstract]) OR “obese”[Title/Abstract]) OR “adipose tissue”[Title/Abstract]) OR “adiposity”[Title/Abstract]) OR “overweight”[Title/Abstract]))) OR “body composition”[Title/Abstract])) AND ((((((“Inositol”[Mesh]) OR “myo‐inositol”[Title/Abstract]) OR “inositol”[Title/Abstract]) OR “inositol phosphate”[Title/Abstract])) OR “d‐chiro‐inositol”[Title/Abstract]).

Relevant publications were identified, and their related articles and citations were also scanned. Besides, the reference lists of all identified articles were examined to avoid missing any eligible study.

### 2.2 Study selection and inclusion and exclusion criteria

Initially based on the PICO (Participants = adults, Intervention = inositol supplementation, Comparison = placebo or control group, Outcome = Changes in body mass index), the following principles were used to include studies: (i) controlled clinical trials with either parallel or cross‐over design with inositol supplementation in adults (ii) investigation of the effects of any type of inositol on BMI, (iii) providing sufficient information at the baseline and end of the trial on BMI in both inositol and control groups. Studies were excluded if they: (i) were observational studies, (ii) were studies without control group, (iii) supplemented inositol concomitant with other ingredients, and (iv) were conducted on pregnant or lactating women.

### 2.3 Data extraction

Data were extracted from eligible studies by two independent reviewers (Azadeh Dehghani and Nima Radkhah) using a specially developed data extraction form according to the selection criteria. The extracted information included the description of the study, participants, intervention (dose and duration of supplementation with inositol) and study results based on the determined aforementioned outcomes. In terms of insufficient information, authors were requested by an e‐mail for providing numerical data and further explanation in the case of any ambiguity, if possible. Any disagreements and doubts were resolved based on consensus discussions with the third reviewer (Meysam Zarezadeh). All extracted data were reappraised by the senior authors.

### 2.4 Quality assessment

Cochrane Collaboration's risk of bias tool was employed to assess bias in the included studies, comprising inadequacy of sequence generation, improper blinding and allocation concealment, not addressing the dropouts (incomplete outcome data), selective outcome reporting, and other potential sources of bias. Based on the Cochrane Handbook recommendations, a judgment of “yes” indicated a low risk of bias, while “no” indicated a high risk of bias. Labeling an item as “unclear” indicated an unclear or unknown risk of bias. Two reviewers (Azadeh Dehghani and Amir Hossein Faghfouri) evaluated the quality of included studies and any discrepancy was resolved through consensus with third reviewer (Meysam Zarezadeh).

### 2.5 Statistical analysis

The current meta‐analysis was performed based on a random‐effects model using restricted maximum likelihood method (REML) if the amount of heterogeneity was high. The *I* ^2^ statistics and Cochrane Q test were used to assess the heterogeneity in which *I* ^2^ > 50% and *p* < 0.1 were defined as high between‐study heterogeneity.^23^ If the amount of heterogeneity was not significant, fixed‐effects model was applied. Effect size was estimated following meta‐analysis of mean differences (MD), and their respective standard deviations (SD). In the cases that mean ± SE, median (range) and median (Q25–Q75) were reported, mean ± SD was estimated using appropriate statistical equations. Due to the identical scale (kg/m^2^) across studies, effect size was presented as weighted mean difference (WMD) as the units of studies variable were identical. Impact of moderator variables including mean age of participants, dosage, duration of supplementation, and sample size on the final effect size was evaluated using meta‐regression analysis based on the presence of any linear relationship. In addition, subgroup analyses were conducted based on the duration of the supplementation, BMI at the baseline, mean age of the study participants, inositol dosage and population groups to clarify the potential sources of heterogeneity and the different diagnoses of the related complications. Sensitivity analysis was performed to assess the influence of each study removal on the overall effect size using leave‐one‐out method. For evaluation of small study effect, Begg's adjusted rank correlation and Egger's regression asymmetry tests were performed.^24^, ^25^ Moreover, publication bias was assessed based on the presence of asymmetry in funnel plot. For adjusting publication bias, trim and fill analysis was carried out with correcting funnel plot asymmetry. The value of *p* < 0.05 was considered as significant level. All statistical analyses were performed using Stata 16.0 (Stata Corporation).

## 3 RESULTS

### 3.1 Study selection

**FIGURE 1:**
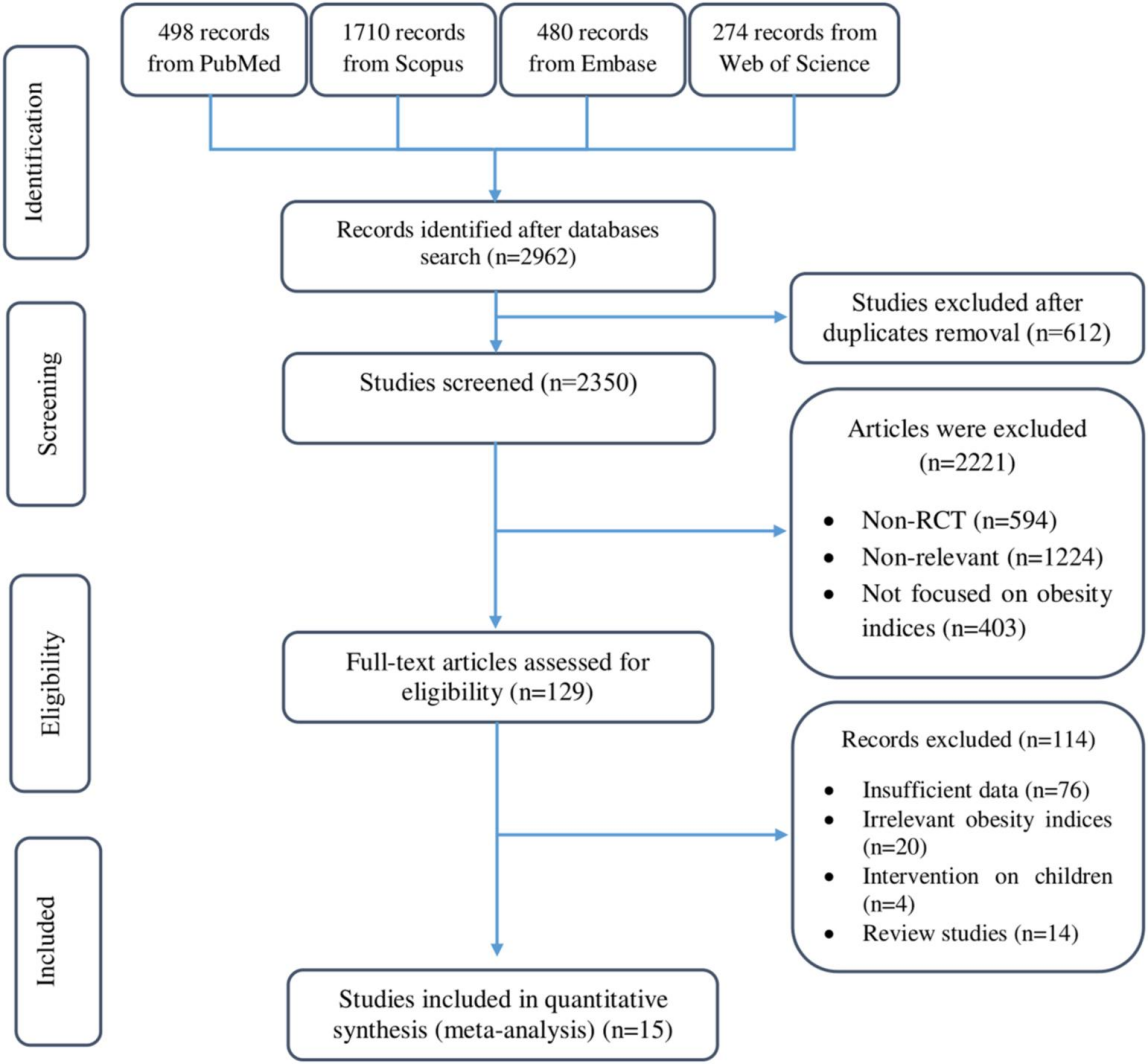
Study selection process demonstrated by PRISMA flow chart

A total number of 2703 citations were obtained from databases among which 2113 articles were screened by titles and abstracts by two independent reviewers (Azadeh Dehghani and Amir Hossein Faghfouri) after excluding 590 duplicate articles. After removing irrelevant articles, full‐text of 125 studies were evaluated. Only studies on the effect of inositol supplementation on BMI scores were selected and reviewed. The number of studies investigating other anthropometric indices was not sufficient to include in data synthesis. Finally, 15 controlled clinical trials were recognized as eligible. The process of study selection is shown in PRISMA flow chart of the study (Figure 1).

### 3.2 Study characteristics

Studies were published between 1999 and 2019. The total number of study participants were 891, of which 48 were male and 843 were female. Mean age of the participants in both arms of intervention and control was between 23.5 ± 2.1 and 55.6 ± 3.2 years. The dosage range of inositol supplementation was 600–4450 mg/day administered for 6–48 weeks. Ten and two of the 15 included studies investigated the effects of inositol supplementation on the patients with PCOS and metabolic syndrome, respectively. The remaining three studies were on pre‐diabetic, diabetic, and healthy participants. Ten studies were performed in Italy,^19^, ^26^, ^27^, ^28^, ^29^, ^30^, ^31^, ^32^, ^33^, ^34^ two in Venezuela,^18^, ^35^ two in Spain,^36^, ^37^ and one in India.^38^ Characteristics of included studies were outlined in Table 1.

**TABLE 1:** Characteristics and baseline measurements of included studies

First author	Location	Year	Patient features	Gender	Sample size (*n*)	INT mean age (year)	CONT mean age (year)	Duration (week)	Type of inositol	Inositol dosage (mg/day)	Baseline BMI (kg/m^2^)	
											INT	CONT
Maurizi, A. R.	Italy	2016	Patients with T1DM	Both	26	35.5 ± 10.0	35.5 ± 10.0	24	D‐chiro inositol	1000	26.2 ± 2.1	27.1 ± 1.2
Agrawal, A.	India	2019	Women with PCOS undergoing ovulation induction cycles	F	120	28.35 ± 2.74	28.35 ± 2.74	12	Myo‐inositol	1800	27.71 ± 3.6	27.38 ± 3.9
Genazzani, A. D.	Italy	2019	Patients with PCOS and overweight/obesity	F	52	NR	NR	12	Myo‐inositol	1000	29.8 ± 7.9	32.8 ± 8.3
Genazzani, A. D.	Italy	2019	Patients with PCOS and overweight/obesity	F	28	NR	NR	12	Myo‐inositol	1000	28.8 ± 4	30.1 ± 10.4
Genazzani, A. D.	Italy	2019	Patients with PCOS and overweight/obesity with familial diabetes	F	24	NR	NR	12	Myo‐inositol	1000	31.6 ± 7.6	34.5 ± 3.8
Iuorno, M. J.	Venezuela	2002	Lean women with PCOS	F	20	28.2 ± 1.5	28.2 ± 1.5	6–8	D‐chiro inositol	600	22.4 ± 0.3	22.1 ± 0.3
Artini, P.G.	Italy	2013	Patients with PCOS	F	50	34.9 ± 2.1	34.9 ± 2.1	12	Myo‐inositol	2000	28 ± 1.6	26.6 ± 2.1
Minozzi, M.	Italy	2011	Patients with PCOS	F	155	28.8 ± 3.8	28.8 ± 3.8	48	Myo‐inositol	4000	26.7 ± 2.8	26.2 ± 2.6
Dona`, G.	Italy	2012	Patients with PCOS	F	26	23.5 ± 2.1	23.5 ± 2.1	12	Myo‐inositol	1200	21.6 ± 1.9	21.9 ± 0.6
Costantino, D.	Italy	2009	Patients with PCOS	F	42	28.8 ± 1.5	28.8 ± 1.5	12–16	Myo‐inositol	4000	22.8 ± 0.3	22.5 ± 0.3
Genazanni, A. D.	Italy	2008	Patients with PCOS and overweight	F	20	NR	NR	12	Myo‐inositol	2000	29 ± 1.6	27.8 ± 2.1
Nestler, J. E.	Venezuela	1999	Patients with PCOS	F	44	29 ± 6	29 ± 6	6–8	D‐chiro inositol	1200	31.3 ± 2.4	31 ± 2.2
Gerli, S.	Italy	2007	Patients with PCOS	F	92	29	29	14	Myo‐inositol	4000	34	34.8
Giordano, D.	Italy	2011	Postmenopausal women with MetS	F	80	55.6 ± 3.2	55.6 ± 3.2	24	Myo‐inositol	2000	31.5 ± 2.4	30.7 ± 2.5
Santamaria, A.	Italy	2012	Postmenopausal women with MetS	F	80	55.6 ± 3.2	55.6 ± 3.2	48	Myo‐inositol	2000	31.5 ± 2.4	30.7 ± 2.5
Bañuls, C.	Spain	2016	Participants with prediabetes	Both	22	53.4 ± 10.7	53.4 ± 10.7	12	Pinitol	4450	26.8 ± 2.4	27.1 ± 2.2
Bañuls, C.	Spain	2016	Middle‐aged participants with prediabetes and obesity	Both	22	53.5 ± 14.5	53.5 ± 14.5	12	Pinitol	4450	35.4 ± 2.8	34.4 ± 3.5
Bañuls, C.	Spain	2016	Middle‐aged participants with prediabetes	Both	44	53.5 ± 12.5	53.5 ± 12.5	12	Pinitol	4450	31.3 ± 5.1	30.7 ± 4.7
Bañuls, C.	Spain	2015	Healthy participants	Both	40	34.0 ± 10.9	34.0 ± 10.9	12	Pinitol	4450	23.8 ± 3.2	24.2 ± 3.5

*Note*: Values are expressed as mean ± SD.

Abbreviations: BMI, body mass index; CONT, Control; F, Female; INT, Intervention; MetS, Metabolic Syndrome; NR, Not Reported; PCOS, Polycystic Ovary Syndrome; T1DM, Type 1 Diabetes Mellitus.

### 3.3 Risk of bias assessment

**FIGURE 2:**
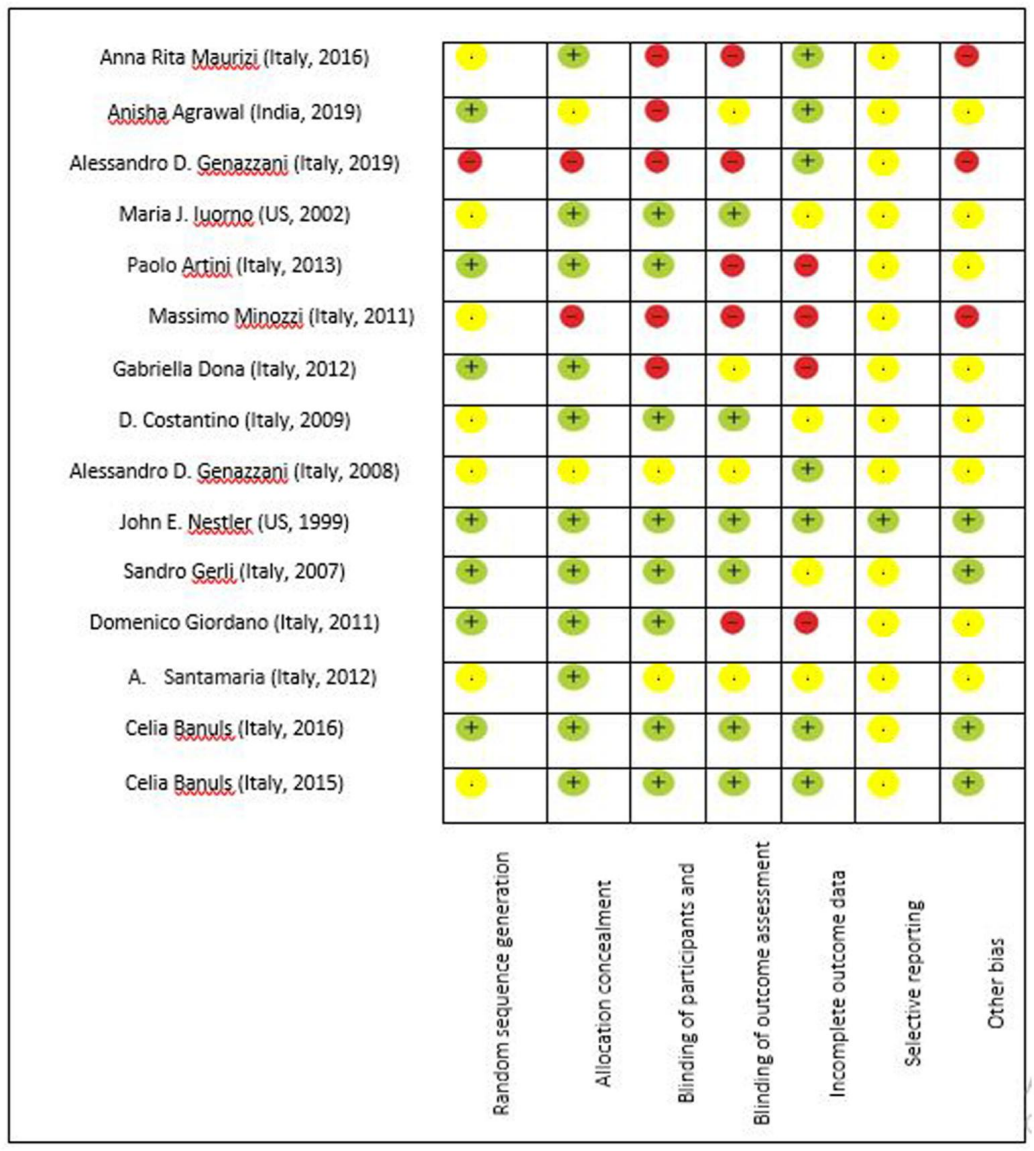
The results of quality assessment of included studies using Cochrane Collaboration's risk of bias tool

According to Cochrane handbook, the results of quality assessment of included studies are presented in Figure 2.

### 3.4 Effect of inositol on BMI

**FIGURE 3:**
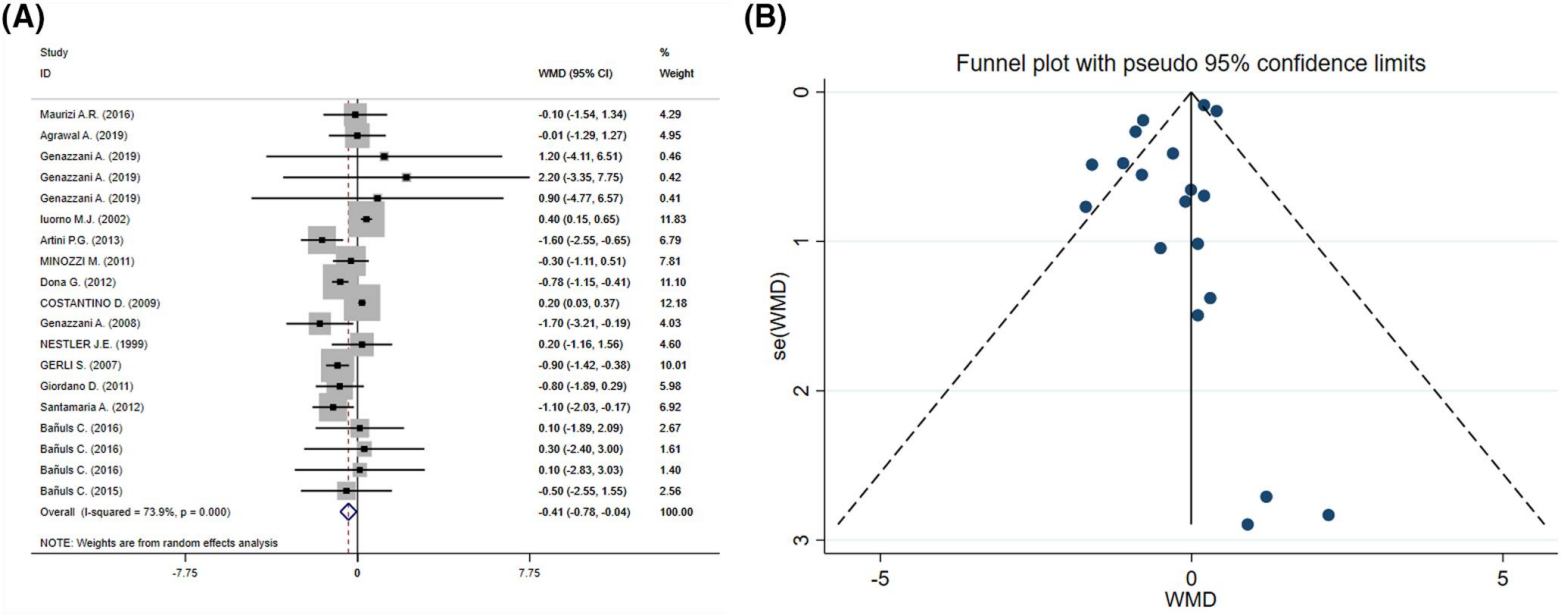
The forest plot (A) and funnel plot (B) of effect of inositol supplementation on body mass index (BMI)

The results of pooled meta‐analysis showed that inositol supplementation significantly decreased BMI scores (WMD = −0.41 kg/m^2^; 95% CI: −0.78, −0.04; *p* = 0.028, *I* ^2^ = 73.9%, *p* < 0.001) (Figure 3A). Duration of supplementation, BMI at the baseline, mean age of participants, type of inositol supplement, and administered dosage and population groups were identified as potential sources of heterogeneity following subgroup analyses (Table 2). In addition, based on subgroup analysis, inositol supplementation had more diminishing effect on BMI scores in individuals with overweight and obesity (Table 2). In addition, it seems that MI supplements has stronger lowering effect on BMI compared with other forms of inositol (Table 2). Moreover, the diminishing effect of inositol on BMI was more considerable in participants with PCOS with overweight and obesity (Table 2). Additionally, Subgroup analysis revealed that intervention for less than 12 weeks and at a dosage <1000 mg had more clinically significant effect. Supplementation in participants with age >40 was more efficacious (Table 2).

**TABLE 2:** Pooled estimates of inositol supplementation effects on BMI within different subgroups

Group	No. of comparisons	WMD (95% CI)	*p*‐value	*I* ^2^ (%)	P‐heterogeneity
Total	19	−0.41 (−0.78, −0.04)	0.028	73.9	<0.001
Inositol dosage (mg)					
≤1000	5	0.39 (0.15, 0.64)	0.002	0.0	0.913
1000–2000	5	−0.81 (−1.42, −0.21)	0.008	47.5	0.106
>2000	9	−0.43 (−0.96, 0.11)	0.117	68	0.002
Intervention duration (week)					
<10	2	0.39 (0.15, 0.64)	0.002	0.0	0.777
12	11	−0.79 (−1.1, −0.48)	<0.001	0.0	0.492
12–24	4	−0.38 (−1.14, 0.38)	0.329	83.5	<0.001
>24	2	−0.66 (−1.44, 0.12)	0.096	38.2	<0.203
Mean age (year)					
<30	11	−0.25 (−0.67, 0.18)	0.255	79.9	<0.001
30–40	3	−0.91 (−1.94, 0.11)	0.081	38.2	0.198
>40	5	−0.74 (−1.38, −0.11)	0.021	0.0	0.708
Baseline BMI (kg/m^2^)					
18.5–25	4	−0.06 (−0.59, 0.46)	0.815	89.6	<0.001
25–30	8	−0.61 (−1.23, 0.02)	0.056	29.7	0.191
>30	7	−0.78 (−1.17, −0.39)	<0.001	0.0	0.684
Type of inositol supplement					
D‐chiro inositol	3	0.38 (0.14, 0.62)	0.002	0.0	0.771
Myo‐inositol	12	−0.66 (−1.16, −0.15)	0.011	79.9	<0.001
Pinitol	4	−0.06 (−1.21, 1.1)	0.925	0.0	0.963
Population groups					
Overweight/obese diabetic	4	0.03 (‐0.98, 1.03)	0.954	0.00	0.994
Overweight/obese PCOS	11	−0.78 (−1.15, −0.41)	<0.001	14.2	0.309
Non‐obese PCOS	3	−0.04 (−0.59, 0.51)	0.893	93.0	<0.001
Healthy participants	1	−0.50 (−2.55, 1.55)	0.028	‐	‐

Abbreviations: BMI, body mass index; PCOS, polycystic ovary syndrome; WMD, weighted mean difference.

Meta‐regression analysis showed that moderator variables including mean age of participants, administered dosage, duration, and sample size had no significant impact on the effect size of the study. No significant difference was observed in results performing sensitivity analysis. There was no small study effect using Egger's test (*p* = 0.114), while Begg's test showed a borderline small study effect (*p* = 0.042). Asymmetry was found in funnel plot based on visual inspection (Figure 3B). However, the same significant result was observed following trim and fill analysis (WMD = −0.41 kg/m^2^; 95% CI: −0.78, −0.04; *p* = 0.028).

## 4 DISCUSSION

In the meta‐analysis of 15 controlled clinical trials, inositol supplementation was associated with reductions in BMI. Subgroup analysis showed that intervention for less than 12 weeks and at a dosage <1000 mg had more clinically significant effect. Supplementation is more effective in people over 40 years of age than in other age groups. Our results showed that inositol supplementation was more effective in people with PCOS and overweight/obesity than in other populations. Among the various types of inositol supplements, MI was the most effective form in lowering BMI.

The results highlight the advantageous effect of MI in ameliorating the metabolic profile of women with PCOS, concomitantly diminishing their hyper‐androgenism.^39^ Celia Bañuls et al. showed that chronic consumption of an inositol‐enriched beverage (IEB) led to a decline in insulin resistance and plasma apolipoprotein B concentrations and an increase in low‐density lipoprotein particle size, despite the lack of changes in serum triglyceride levels. Moreover, chronic IEB consumption significantly improved carbohydrate metabolism parameters in healthy participants.^36^ These findings underlined the efficacy of inositol supplementation as an initiative strategy to attenuate hyperglycemia and other morbidities caused by insulin resistance as well as risk of cardiovascular disease. Another study indicated that consumption of IEB for 12 weeks led to inconsistent response in pre‐diabetic patients depending on BMI, with an improvement in insulin resistance as well as postprandial and nocturnal glycemic control in people without obesity and an anti‐inflammatory response in individuals with obesity.^37^

In a short three‐armed study conducted by Chirania et al., the effects of metformin (1500 mg), MI (1 g/day), or the mixture of both were compared in infertile women with PCOS. There were remarkably further improvements in symptoms and hormonal parameters, and weight loss in both the second and third groups. The conclusion was that MI could be suggested for women with PCOS.^40^ Of note, DCI supplementation was also associated with a decreases in diastolic and systolic blood pressures, serum levels of triglyceride, total cholesterol and circulating insulin, improved glucose tolerance and reduced serum androgen concentrations.^18^ Remarkably, inositol has been characterized as a second messenger system resulting in enhanced metabolic enzymes activity due to its insulin‐like properties.^19^, ^41^

Regarding possible mechanisms, as previously articles stated, DCI oral supplementation may ameliorate glucose metabolism and partially restore peripheral insulin sensitivity^42^ and oocyte quality, decrease hyper‐androgenism and adjust menstrual cycles ovulation and hirsutism.^39^, ^43^ GLUT4, an insulin‐sensitive glucose transporter, has a well‐known role in controlling insulin‐motivated glucose transport into skeletal muscle and adipose tissue, so that connecting insulin to its receptor leads the translocation of GLUT4 from intracellular storage vesicles to the cellular surface via protein phosphorylation cascade through the phosphatidylinositol‐3 kinase/Akt signaling pathway.^44^, ^45^, ^46^ An imperfection in glucose transport efficiency, containing GLUT4 expression and function, results in insulin resistance.^47^ The benefits of an oral chemically synthesized PI for lowering plasma glucose level, increasing insulin sensitivity and boosting glucose disposal capacity has attracted a lot of attention in experimental animals and humans with impaired glucose metabolism.^42^, ^48^, ^49^

There was a significant between‐study heterogeneity in the present study which might overshadow the conclusions, however, after subgroup analysis, inositol dosage (mg), intervention duration (Week), mean age (Year), baseline BMI (kg/m^2^), type of inositol supplement, and population groups were identified as source of heterogeneity which validate the findings and conclusions. Subgroup analysis showed that supplementation was more effective in the short term of less than 12 weeks and at the dosage of less than 1000 mg. One possible explanation is the fact that inositol, as postprandial and nocturnal glycemic control, is more effective in the shorter time and is not effective in the long term due to changes in glucose and fat levels or their mechanisms.^50^, ^51^, ^52^ In addition, more sensible positive effect of inositol in individuals suffering PCOS, might be due to higher levels of fat and hyper‐androgenism.^53^, ^54^ Regarding its greater effectiveness at the age >40, it might be due to its effect on increasing lipoprotein particle size.^43^, ^55^, ^56^

Although the overall results of the present study showed that the effect of inositol in lowering BMI was statistically significant, however, the magnitude of this effect is clinically small and may not be considered as a single therapeutic approach. Nevertheless, complementary therapy of inositol along with other interventions such as weight loss diets or energy‐restricted diets or dietary supplements and exercise, could result in robust clinical impact on the anthropometric indices, especially BMI.

Regarding the therapeutic use of inositol, including in diseases such as PCOS, it should be noted that in terms of safety and possible side effects, only the highest dose of MI (12 g/day) causes mild gastrointestinal side effects such as nausea, bloating, and diarrhea. In addition, the intensity of harmful events stays also the similar at 30 g/day. Remarkably the dosage of 4 g/day of inositol generally used in clinics is entirely free of side effects.^7^

To our knowledge, present study is the first systematic review and meta‐analysis summarizing the effect of inositol supplementation on BMI, however, it had some limitations. First, the overall sample size of study was small which could theoretically impact on estimates of treatment effect. Second, due to the small number of available experiments, other anthropometric indicators such as weight and waist circumference were not considered in the meta‐analysis. Third, only articles in English have been included in the study. Fourth, differences in dosage and study durations of included studies might lead to different findings. Fifth, high between‐study heterogeneity could be considered as one of the limitations of the study, although the sources of it were identified. Also, participants were of different races, and this may play a role in the effect of inositol, which this results did not consider racial and genetic differences.

## 5 CONCLUSION

This study elucidated the significant reducing effect of inositol on BMI in adults younger than 30 years as well as in individuals with PCOS, although the clinical magnitude of this effect may not be much considerable. However, inositol supplementation could be administered as an adjunct therapy to improve anthropometric indices and glycemic responses.

## AUTHOR CONTRIBUTIONS

Meysam Zarezadeh : Contributions to concept/design, Data analysis/interpretation, Critical revision of the manuscript, Approval of the article; Azadeh Dehghani : Drafting of the manuscript, Acquisition of data, Approval of the article; Amir Hossein Faghfouri : Drafting of the manuscript, Acquisition of data, Approval of the article; Nima Radkhah : Acquisition of data, Approval of the article; Mohammad Naemi Kermanshahi: Drafting of the manuscript, Approval of the article; Niyaz Mohammadzadeh Honarvar : Acquisition of data, Approval of the article; Zohreh Ghoreishi: Critical revision of the manuscript, Approval of the article; Alireza Ostadrahimi : Critical revision of the manuscript, Approval of the article.

## CONFLICT OF INTEREST

The authors declare no conflicts of interest.
